# Normed dataset for novel metaphors, novel similes, literal and anomalous sentences in Chinese

**DOI:** 10.3389/fpsyg.2022.922722

**Published:** 2022-09-01

**Authors:** Xin Wang

**Affiliations:** College of Foreign Languages, Henan University, Kaifeng, China

**Keywords:** normed dataset, novel metaphors, novel similes, literal sentences, anomalous sentences

## Introduction

Metaphors are pervasive in daily communication. In uttering a metaphor, the speaker usually means something different from what he or she literally says (Gibbs and Colston, 2012). For example, in saying “*My lawyer is a shark*,” the speaker may intend to communicate “*My lawyer is ferocious*” other than its literal meaning “*My lawyer is a marine animal*.” Investigating how metaphors are comprehended is one of the main concerns of psycholinguistics (Bambini et al., 2014). However, this question still remains unresolved in the literature. The Standard Pragmatic Model (Grice, 1975) suggests that a metaphorical meaning comes after a literal interpretation. It also suggests that similes would be easier to understand than metaphors. In contrast, the Direct Access Model (Gibbs, 1994) claims that a figurative meaning is accessed directly without rejection of a literal meaning first. Moreover, metaphors are no more difficult to comprehend than similes (Tartter et al., 2002). Other metaphor theories such as the Graded Salience Hypothesis (Giora, 2003) holds that the most salient meaning, namely, the most frequent, familiar, conventional and prototypical meaning is accessed initially. There are conflicting findings from experimental studies of metaphor comprehension, and all these models find some support. For example, Ashby et al. (2018) used an eye tracking method to examine how people read metaphors and similes. The results found that metaphors required longer reading time as compared to similes, indicating that metaphors were harder to process than similes. Such results were interpreted as supporting the view that readers initially hold one primary interpretation. There are many reasons for these conflicting findings, some being methodological and some being theoretical (De Grauwe et al., 2010, p. 1,967). Methodologically, for example, some studies failed to control the frequency and concreteness of the critical words, or familiarity of the sentence stimuli. Therefore, contemporary psycholinguistic studies have devoted a great effort to controlling the variables that might impact metaphor comprehension. To the best of our knowledge, Katz et al. (1988) pioneered this field by presenting norms for 464 metaphors on 10 dimensions: comprehensibility, ease of interpretation, metaphoricity, metaphor goodness, metaphor imagery, subject or tenor imagery, predicate or vehicle imagery, felt familiarity, semantic relatedness and number of alternative interpretations. Among the 464 metaphors, 204 were literary metaphors (e.g., *Mankind is a cripple whose stick taps through horror-filled dreams*) and 260 were nonliterary metaphors, with either simple topics and vehicles (e.g., *Freedom is truth*) or complex topics and vehicles (e.g., *A white rabbit's fur in winter is a ready-made suit of long underwear*). Cardillo et al. (2010) presented a normed dataset for 280 metaphorical and 280 literal sentences along 10 dimensions on word level (length, frequency, concreteness) and on sentence level (familiarity, naturalness, imageability, figurativeness, interpretability, valence and valence judgement reaction time). The stimuli were either nominal (e.g., Metaphorical: *The marriage was a long sob*; Literal: *The sound was a bitter sob*) or predicative (e.g., Metaphorical: *The hard candy rattled in the box*; Literal: *The violent image rattled in her head*). In addition, Roncero and de Almeida (2015) developed norms for 84 topic-vehicle pairs, which were written as copular metaphors/similes (e.g., *Exams are/ are like hurdles*), isolated topic (e.g., *exam*) or vehicle words (e.g., *hurdles*). The datasets were well controlled for in terms of properties, familiarity, aptness, conventionality, connotativeness and interpretive diversity. More recently, Jankowiak (2020) created 480 stimuli of novel metaphors, novel similes, literal and anomalous sentences in Polish and English. The stimuli were thoroughly normed along the dimensions of a number of variables: meaningfulness, familiarity, metaphoricity, and level of predictability. However, the aforementioned normed dataset mainly focused on metaphor use in alphabetic languages, especially in English, while previous research into semantic processing has indicated that cognitive mechanisms engaged in lexico-semantic access might be sensitive to language-specific characteristics (Cheng and Caldwell-Harris, 2010). With a view to filling this gap, the present paper was devoted to providing the first dataset of novel metaphors, novel similes, literal and anomalous sentences in Chinese that have been extensively normed both at the word and sentence level.

In essence, metaphor is used to understand one entity (the target domain) in terms of another (the source domain) (Lakoff and Johnson, 1980). Metaphor comprehension is hypothesized to require the structural alignment of the source domain and the target domain (Bowdle and Gentner, 2005) or property attribution, by which properties of the source domain are attributed to the target domain (Glucksberg and Keysar, 1990). As a consequence, generally, metaphors, especially novel (unfamiliar) metaphors demand more cognitive effort to process than literal sentences, indicative of the semantic incongruity between the source domain and the target domain (Coulson and Van Petten, 2002; Arzouan et al., 2007; Obert et al., 2018; Jankowiak, 2020; Jankowiak et al., 2021; Wang and Jankowiak, 2021). Importantly, according to the Career of Metaphor Model (Bowdle and Gentner, 2005), metaphor processing is modulated by its level of conventionality. A conventional metaphor (e.g., *My job is* a jail) involves a source domain that refers both to a literal referent (e.g., *a jail*) and to a domain-general category (e.g., *a confining place*). Conventional metaphors may therefore be interpreted as categorizations in which the target domain is regarded as a member of a superordinate category. In contrast, a novel metaphor involves a source domain that only refers to a literal, domain-specific referent. The Career of Metaphor Model further postulates that a novel metaphor (e.g., *Science is a glacier*) is easier and faster to comprehend when in the form of a simile (e.g., *Science is like a glacier*), for the reason that similes automatically initiate comparison mechanisms engaged in novel meaning processing (Jankowiak, 2020). This hypothesis has been tested in a number of studies, in which behavioral, functional magnetic resonance imaging (fMRI) or event-related potential (ERP) methods were employed (Bowdle and Gentner, 2005; Shibata et al., 2012; Lai and Curran, 2013; Jankowiak et al., 2021). For example, Bowdle and Gentner (2005) observed that novel similes required shorter comprehension time than novel metaphors, indicative of the facilitating effect of a simile form in novel metaphor comprehension. In an ERP study by Lai and Curran (2013) (experiment 2), the researchers primed novel metaphoric sentences (e.g., *Ideas can sometimes be bumpy*) with related similes (e.g., *Ideas are like roads*) and found that simile primes reduced the N400 differences between novel metaphors and literal sentences, thus indicating that comparison mechanism facilitated the comprehension of novel metaphors. More recently, in another ERP study, Jankowiak et al. (2021) compared the processing of novel metaphors (e.g., *Memory is a bag*), novel similes (e.g., *Memory is like a bag*), literal sentences (e.g., *This package is a bag*) and anomalous sentences (e.g., *Screen is a bag*) in both L1 (Polish) and L2 (English) among highly proficient Polish-English bilingual speakers. The results showed that within the N400 time window, novel similes facilitated the processing of novel metaphors only in the native language. Additionally, within the LPC (Late Positive Complex) time window, the authors observed the facilitating effect of novel similes in both the native language and the non-native language. Taken together, these studies show that the linguistic form of novel similes automatically initiates comparison processes, which might ease novel meaning comprehension (Jankowiak et al., 2021).

However, thus far little attention has been devoted to testing the hypotheses of the Career of Metaphor Model in non-alphabetic languages, such as Chinese. As a consequence, it remains unclear whether a comparison structure in similes facilitates novel metaphoric meaning comprehension in Chinese, especially given that in Chinese, novel metaphors and novel similes share similar morphosyntactic characteristics. To be specific, in Chinese, metaphors, such as “年龄是闹钟” (Eng. *Age is an alarm clock*) and similes, such as “年龄像闹钟” (Eng. *Age is like an alarm clock*) both “是” (Eng. *is*) in metaphors and “像” (Eng. *is like*) in similes perform the function of a verb. As a result, novel similes novel metaphors are very much alike in terms of morphosyntactic features. In order to shed light on this aspect, the present study is aimed at investigating whether comparison mechanisms activated by a form of a simile facilitate novel meaning construction in Chinese.

It is noteworthy that several studies have indicated that apart from metaphor conventionality, other factors of the stimuli, such as meaningfulness, familiarity, metaphoricity, and cloze probability could also mediate metaphoric meaning processing (De Grauwe et al., 2010; Jankowiak, 2020; Jankowiak et al., 2021). Familiarity reflects how frequently a language user encounters a particular expression (Libben and Titone, 2008; Bosco et al., 2012; Bambini et al., 2014; Jankowiak, 2020). It is a frequently addressed parameter in metaphor processing (e.g., Connine et al., 1990; Schmidt and Seger, 2009). Since unfamiliar metaphors are usually novel or unconventional, familiarity is often used interchangeably with conventionality (Cardillo et al., 2010; Jankowiak et al., 2017). Meaningfulness refers to whether the sense of the utterances is interpretable (Bambini et al., 2016). It also measures whether the participants understand (or not) the expressions. Metaphoricity evaluates the degree to which a sentence is interpreted metaphorically (Yang et al., 2013). In studies on metaphor processing, studies have revealed significantly higher metaphoricity of metaphorical sentences relative to literal sentences (Yang et al., 2013; Jankowiak, 2020; Jankowiak et al., 2021; Wang and Jankowiak, 2021). Cloze probability assesses the extent to which a particular word is expected due to the preceding context (Kutas and Federmeier, 2011; Bambini et al., 2014, 2016). Studies indicated that cloze probability for metaphors, especially for novel metaphors is much lower than for literal sentences (Coulson and Van Petten, 2002; Jankowiak et al., 2017; Obert et al., 2018).

The present study is devoted to examining the meaningfulness, familiarity, metaphoricity and cloze probability of novel metaphors, novel similes, literal and anomalous sentences in Chinese by testing all of the above-mentioned variables. Basic definitions of these variables and an overview of the rating tasks we conducted are provided in Table 1. Importantly, as in Chinese novel metaphors and novel similes are very much similar in terms of their morphosyntactic features, by testing these variables, the present study offers a new perspective on whether comparison processes initiated by similes facilitate novel meaning comprehension in non-alphabetic languages. Additionally, the present study aims to provide a dataset of novel metaphors, novel similes, literal and anomalous sentences in Chinese so as to test new and existing models of metaphor comprehension.

**Table 1:** Basic definitions of the variables and an overview of the rating tasks.

**Task, variable**	**Variable definition**	**Instruction summary**	**Sample item**	**Items (*N*)**	**Participants (*N*)**
Critical words' concreteness	Whether a word can be used as the object of a sense verb (e.g., touch, see, hear etc.)	根据1-7级量表,请判断以下名词的具体等级 (Eng. *Rate from 1 to 7 how concrete a noun is.)*	憎恨 (Eng. *hatred*), 桌子 (Eng. *table*)	120	31
Cloze probability	The extent to which a word is expected due to the preceding context	对于下面每一个句子,请用你最先想到的一个名词将之补充完整,并保证句子通顺、有意义。 (Eng. *Add a noun that first comes to your mind to the presented beginning of a sentence, so that the sentence is semantically meaningful and grammatically correct*.)	秋天是 ____. (Eng. *Autumn is ____*.) 太阳像. (Eng. *The Sun is like ____*) 这个猴子是 (Eng. *This monkey is a (n) ____*	360	157
Meaningfulness	Whether the sense of the utterances is interpretable	根据1-7级量表,请判断以下句子的意义度 (Eng. *Rate from 1* *to 7 how meaningful a sentence is*.)	读者是猎手 (Eng. *Readers are hunters*.)祖国像卫兵(Eng. *The motherland is like a guard)*. 这群鸟是天鹅。(Eng. *These birds are swans*.) 国旗是蒸汽。(Eng. *The national flag is steam*.)	480	108
Familiarity	How frequently a participant encounters an expression	根据1-7级量表,请你判断碰到以下句子的经常性 (Eng. *Rate from 1* *to 7 how often you encounter a sentence.)*	危机是传染病(Eng. *A crisis is an infectious disease*.) 外科医生像木匠。(Eng. *Surgeons are like carpenters*.) 那个人是逃兵(Eng. *That man is a deserter*.)	360	88
Metaphoricity	The degree to which a sentence is interpreted metaphorically	根据1-7级量表,请判断以下句子的隐喻度 (Eng. *Rate (from 1* *to 7) how metaphorical or literal a sentence is*.)	死亡是调料(Eng. *Death is a seasoning*.) 时间像磁铁(Eng. *Time is like a magnet*.) 他的叔叔是信使。(Eng. *His uncle is a postman*.)	360	95

Original Chinese, English translations in italics.

## Methods

### Construction of materials

Four hundred and eighty sentences, including 120 novel metaphors (e.g., “欲望是牙膏”, Eng. *Desire is a toothpaste*), 120 novel similes (e.g., “欲望像牙膏”, Eng. *Desire is like a toothpaste*), 120 literal sentences (e.g., “这种日用品是牙膏”, Eng. *This commodity is a toothpaste*), and 120 anomalous sentences (e.g., “泥坑是牙膏”, Eng. *The muddy puddle is a toothpaste*) were employed in the present ratings. The complete stimuli are provided in the Supplementary material.

To generate these sentence types, 120 nouns were first selected as the critical words (sentence-final words). The critical words, which were all concrete nouns were selected from SUBTLEX-CH-WF corpus (Cai and Brysbaert, 2010). The frequency values of the critical words were also calculated using this corpus. The mean frequency per million of the critical words is 3.25 (*SD* = 0.87, range 2–5). Besides, the mean number of characters in the critical words is 2.22 (*SD* = 0.41, range 2–3). Next, for 120 critical words, 120 novel metaphors and 120 novel similes were created. The sentences were selected either from the Chinese poetry network (www.modernchinesepoetry.com) or Center for Chinese Linguistics PKU Corpus (Modern Chinese) (http://ccl.pku.edu.cn:8080/ccl_corpus/) and were adapted, when necessary, to conform to the form of novel metaphor condition and novel simile condition of the present stimuli. The 120 corresponding literal sentences and 120 anomalous sentences were then created. The literal sentences were constructed using semantically compatible items. While novel metaphors and similes were selected and adapted from poetry or novels, the anomalous sentences were created anew to reflect some even greater absurdity (i.e., world knowledge violation) as judged by the experimenter. In this way, there were 120 sentence sets (120 novel metaphors, 120 novel similes, 120 literal sentences and 120 anomalous sentences). Each set shared the same critical word (the sentence-final word). Novel metaphor and novel simile conditions shared the same topic and vehicle, but in contrast to the metaphor condition which uses “是” (Eng. *is*) to relate the two items (the topic and vehicle), the simile condition uses “像” (Eng. *is like*) instead (i.e., *A* 是*B*; Eng: *A is B* vs. *A* 像*B*; Eng: *A is like B*). The number of words per sentence was also controlled for; novel metaphors: *M* = 5.76, *SD* = 0.99, novel similes: *M* = 5.76, *SD* = 0.99, literal sentences: *M* = 6.13, *SD* = 1.08, and anomalous sentences: *M* = 5.54, *SD* = 0.73.

### Overview of norming studies

All the ratings were conducted online using Chinese web-based questionnaires (https://www.wjx.cn/). The stimuli were normed at both word and sentence levels. At the word level, the concreteness values of the critical words were assessed. Concreteness refers to whether a word can be used as “the object of a sense verb (e.g., touch, see, hear etc.)” (Balota et al., 2006, p. 320). Concrete words, such as *table, solider*, and *bread* refer to tangible objects or events, and are more likely to elicit an image of specific referents compared to abstract words, such as *love, failure* and *hope* (Clark and Paivio, 1991, p. 155). Concreteness ratings were assessed to ensure that all the critical words chosen from the corpus were indeed concrete. The concreteness values of critical words were rated by the chosen participants (Norming Study 1). At the sentence level, a different group of participants rated the stimuli in terms of meaningfulness, familiarity, metaphoricity and cloze probability (Norming Study 2). Meaningfulness ratings were administered to ensure that all the presented sentence types, except for anomalous sentences, were perceived as meaningful. Familiarity ratings were aimed to ensure that novel metaphors and novel similes are less familiar than literal sentences. Metaphoricity ratings were aimed to ensure that novel metaphors and novel similes were assessed as metaphorical and literal sentences were evaluated as literal (Jankowiak, 2020). Finally, cloze probability tests were aimed to ensure that the preceding context did not establish an anticipation for an upcoming critical word (Bambini et al., 2014; Jankowiak et al., 2021). To ensure that no critical word appeared more than once within one block, for the meaningfulness ratings, the stimuli were divided into four blocks, and for familiarity ratings, metaphoricity ratings and cloze probability tests, the stimuli were divided into three blocks. Each rater was invited to complete just one block. The stimuli were counterbalanced and the order of presentation within each rating task was randomized. Raters whose scores were more than 3 *SDs* from the mean were removed from the final analyses (Dong et al., 2018; Jankowiak, 2019). Altogether, <4% extreme data were removed (for meaningfulness ratings: 1.82%; for familiarity ratings: 1.12%; for metaphoricity ratings: 1.04%). All the ratings are provided in the Supplementary material.

### Norming study 1: Critical words

#### Ethics statement

The procedures applied in the studies were in accordance with the ethical guidelines for research with human participants, as recommended and followed by Henan University. All participants were informed about the procedures and agreed to participate. All data were collected anonymously.

#### Participants

Thirty-one participants (*M*_age_ = 18.3, *SD* = 0.82; seven males) were recruited from Henan University. They were given monetary compensation for their participation. The participants were all native speakers of Chinese. Participants who failed to complete the whole survey were removed from the final analysis.

#### Stimuli

All the 120 critical words (sentence-final words) were used for the concreteness rating task.

#### Task

Participants were instructed to rate the 120 critical words along with 120 abstract filler words on a 7-point Likert scale from one (very abstract) to seven (very concrete). All the abstract words were selected from Zhang and Lin (1992). These abstract nouns may describe emotions (e.g., “爱情”, Eng. *love*), knowledge (e.g., “经验”, Eng. *experience*), attributes (e.g., “才华”, Eng. *talent*), policies (e.g., “方针”, Eng. *guidelines*), effect (e.g., “成就”, Eng. *achievement*) or other abstract meanings.

Participants were provided with instructions together with a few examples and explanations (see the Supplementary material for the detailed instructions). The rating task took ~10 min to complete.

#### Data analysis

An independent samples *t*-test showed that the critical words (*M* = 5.96, *SD* = 0.43) were evaluated as more concrete than abstract filler items (*M* = 3.10, *SD* = 0.48), *t* (238) = 49.03, 95% CI [2.75, 2.98], *p* < 0.001.

### Norming study 2: Sentences

#### Participants

Altogether, 448 participants who did not participate in the norming study of critical words (Norming Study 1) volunteered in these rating tasks. The participants were recruited from Henan University. They all agreed to participate and were given monetary compensation for their participation. All of the participants were native Chinese speakers, among whom 108 participants (*M*_age_ = 22.52, *SD* = 3.72; nine males) completed the meaningfulness ratings, 88 participants (*M*_age_ = 21.16, *SD* = 2.68; nine males) completed the familiarity ratings, 95 participants (*M*_age_ = 18.77, *SD* = 0.78; 13 males) completed the metaphoricity ratings, and 157 participants (*M*_age_ = 19.24, *SD* = 1.54; 25 males) completed the cloze probability tests. Ethical procedures were as in Norming Study 1.

#### Stimuli

While Meaningfulness ratings were aimed to evaluate whether all the sentence conditions were meaningful or not, familiarity, metaphoricity ratings and cloze probability tests were only aimed to assess meaningful sentences. Consequently, meaningfulness ratings were collected for all the four sentence types: novel metaphors, novel similes, literal and anomalous sentences. Familiarity, metaphoricity ratings and cloze probability tests were only collected for novel metaphors, novel similes and literal sentences.

#### Task

For the meaningfulness ratings task, participants were instructed to rate sentence meaningfulness on a 7-point Likert scale ranging from one (totally meaningless) to seven (totally meaningful). For the familiarity ratings task, raters were asked to rate how familiar they were with the presented sentences on a 7-point Likert scale ranging from one (very unfamiliar) to seven (very familiar). For the metaphoricity rating task, participants were instructed to decide how metaphorical the stimuli were on a 7-point Likert scale ranging from one (totally literal) to seven (totally metaphorical). In addition, for the cloze probability tests, participants were shown a sentence without the critical word (sentence-final word), and were asked to write a noun that first came to their mind so as to make the sentence semantically plausible and syntactically correct. In all the tasks, participants were provided with explanations together with several examples (see the Supplementary material for the detailed instructions). Each rating task lasted ~20 min.

#### Data analysis

To measure the reliability of the rating tests on meaningfulness, familiarity, and metaphoricaity, intraclass correlation coefficients were calculated for all these variables. Results showed that intraclass correlation coefficient for meaningfulness ratings was 0.84, for familiarity ratings was 0.80, and for metaphoricity ratings was 0.89, which suggested a high consistency across raters.

Cloze probability was calculated by dividing the number of raters who completed the sentence fragments with the exact critical words by the total number of raters (Bambini et al., 2014). Mean cloze probability was 0.06% (*SD* = 0.32%) for novel metaphors, 0.08% (*SD* = 0.43%) for novel similes, 3.38% (*SD* = 5.41%) for literal sentences. Besides, for meaningfulness, familiarity and metaphoricity ratings, analyses of variance (*ANOVAs*) were conducted. Significant values for pairwise comparisons were corrected for multiple comparisons using the Bonferroni correction. When Mauchly's tests showed that the assumption of sphericity was violated, the Greenhouse-Geisser correction was applied, and the original degrees of freedom were reported with the corrected *p*-value.

With regard to the meaningfulness ratings, an Analysis of Variance (*ANOVA*) we ran showed a main effect of utterance type, *F*_(3,321)_ = 801.75, *p* < 0.001,ηp2 = 0.882. Pairwise comparisons further revealed that literal sentences [*M* = 5.97, *SE* = 0.09, 95% CI (5.78, 6.16)] were more meaningful than novel similes [*M* = 4.55, *SE* = 0.08, 95% CI (4.39, 4.71)], *p* < 0.001, than novel metaphors [*M* = 4.13, *SE* = 0.07, 95% CI (3.98, 4.27)], *p* < 0.001, as well as than anomalous sentences [*M* = 1.63, *SE* = 0.04, 95% CI (1.56, 1.71)], *p* < 0.001. Additionally, novel similes were rated as more meaningful than novel metaphors, *p* < 0.001, as well as than anomalous sentences, *p* < 0.001. Finally, novel metaphors were evaluated as more meaningful than anomalous sentences, *p* < 0.001.

As for the familiarity ratings, an Analysis of Variance (*ANOVA*) indicated a significant main effect of utterance type, *F*_(2,174)_ = 559.09, *p* < 0.001,ηp2 = 0.865. Pairwise comparisons further revealed that literal sentences [*M* = 5.30, *SE* = 0.11, 95% CI (5.09, 5.52)] were more familiar than both novel similes [*M* = 2.56, *SE* = 0.08, 95% CI (2.40, 2.71)], *p* < 0.001, and novel metaphors [*M* = 2.38, *SE* = 0.08, 95% CI (2.23, 2.53)], *p* < 0.001. Additionally, novel similes were evaluated as more familiar than novel metaphors, *p* < 0.001.

Additionally, as to the metaphoricity ratings, an Analysis of Variance (*ANOVA*) indicated a main effect of the utterance type, *F*_(2,188)_ = 1053.73, *p* < 0.001,ηp2 = 0.918. Pairwise comparisons further revealed that novel metaphors [*M* = 5.95, *SE* = 0.09, 95% CI (5.76, 6.13)] were rated as more metaphorical than novel similes [*M* = 5.25, *SE* = 0.11, 95% CI (5.03, 5.47)], *p* < 0.001, and than literal sentences [*M* = 1.55, *SE* = 0.04, 95% CI (1.46, 1.63)], *p* < 0.001. In addition, novel similes were rated as more metaphorical than literal sentences, *p* < 0.001.

**Figure 1:**
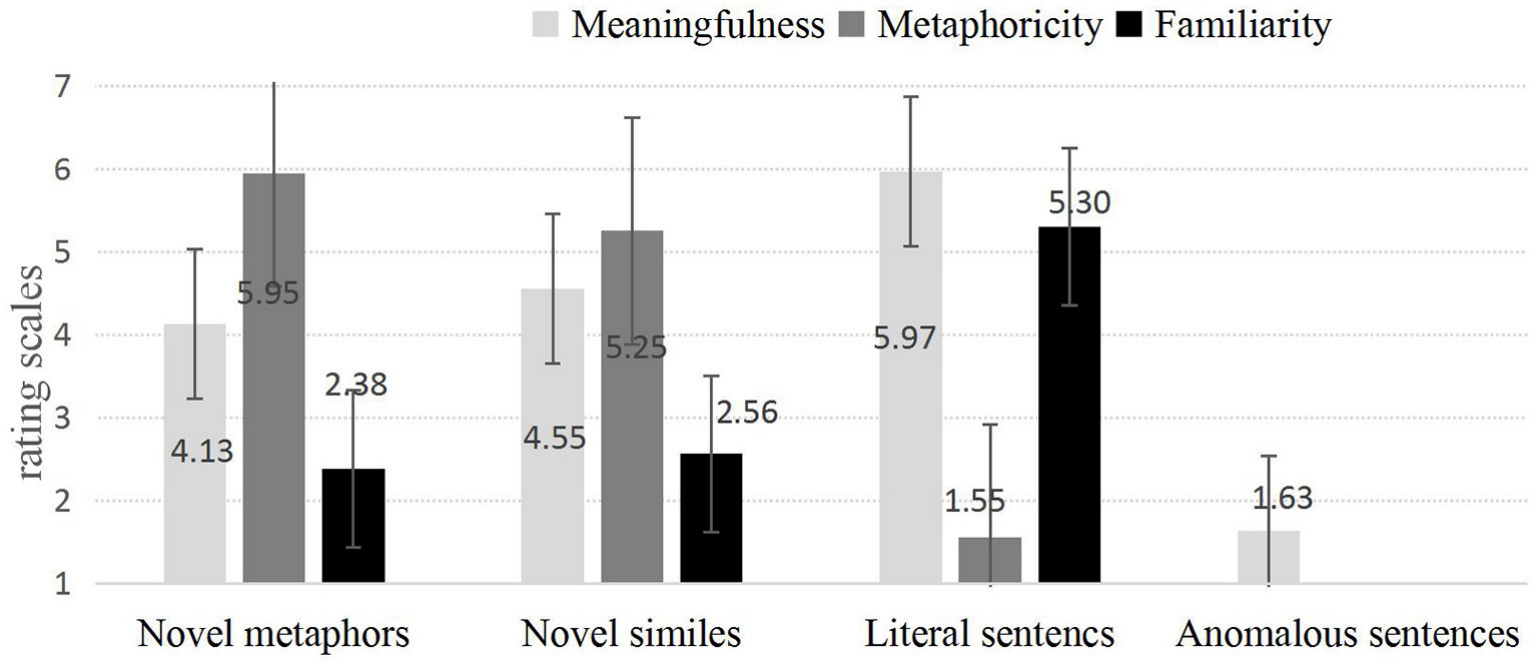
Meaningfulness, familiarity and metaphoricity ratings for novel metaphors, novel similes, literal, and anomalous sentences.

Results for the meaningfulness, familiarity and metaphoricity ratings are presented in Figure 1.

The above norming tests produced the expected differences between all the sentence types. Interscale correlations between cloze probability tests, familiarity, meaningfulness, and metaphoricity ratings are provided in Table 2.

**Table 2:** Interscale correlations between cloze probability tests, familiarity, meaningfulness, and metaphoricity ratings.

	**Cloze probability**	**Familiarity**	**Meaningfulness**	**Metaphoricity**
**Novel metaphors**				
Cloze probability	1	0.071	0.031	−0.108
		*p* > 0.05	*p* > 0.05	*p* > 0.05
Familiarity	0.071	1	0.692^**^	−0.014
	*p* > 0.05	.	*p* < 0.0005	*p* > 0.05
Meaningfulness	0.031	0.692^**^	1	−0.080
	*p* > 0.05	*p* < 0.0005	.	*p* > 0.05
Metaphoricity	−0.108	−0.014	−0.080	1
	*p* > 0.05	*p* > 0.05	*p* > 0.05	.
**Novel similes**				
Cloze probability	1	0.181^*^	−0.025	−0.091
		*p* = 0.047	*p* > 0.05	*p* > 0.05
Familiarity	0.181^*^	1	0.661^**^	−0.277^**^
	*p* = 0.047	.	*p* < 0.0005	*p* = 0.002
Meaningfulness	−0.025	0.661^**^	1	−0.290^**^
	*p* > 0.05	*p* < 0.0005	.	*p* = 0.001
Metaphoricity	−0.091	−0.277^**^	−0.290^**^	1
	*p* > 0.05	*p* = 0.002	*p* = 0.001	.
**Literal sentences**				
Cloze probability	1	0.300^**^	0.145	−0.218^*^
		*p* = 0.001	*p* > 0.05	*p* = 0.017
Familiarity	0.300^**^	1	0.524^**^	−0.167
	*p* = 0.001	.	*p* < 0.0005	*p* > 0.05
Meaningfulness	0.145	0.524^**^	1	−0.195^*^
	*p* > 0.05	*p* < 0.0005	.	*p* = 0.033
Metaphoricity	−0.218^*^	−0.167	−0.195^*^	1
	*p* = 0.017	*p* > 0.05	*p* = 0.033	.

* *p* < 0.05;

** *p* < 0.01.

Finally, as mentioned above, some studies (Bowdle and Gentner, 2005; Shibata et al., 2012; Lai and Curran, 2013; Jankowiak, 2020; Jankowiak et al., 2021) supported the hypothesis of the Career of Metaphor Model (Bowdle and Gentner, 2005) that a form of a simile facilitates the process of meaning creation engaged in novel metaphor processing. However, the previous research was conducted using alphabetic languages, such as Polish where novel metaphors and novel similes differ in their morphosyntactic representations, as a result of which different expectations might have been generated. Consequently, their results may not necessarily be corroborated by a study using novel metaphors, novel similes, literal and anomalous sentences in Chinese. To provide valuable insights into this aspect, we further conducted an online behavioral experiment utilizing our stimuli pool. Unlike offline methods (i.e., questionnaires) which measure how much the participants know, online techniques tend to involve some measure of accuracy and speed, thus being able to provide a clear picture of how real-time processing is carried out (Heredia and Cieślicka, 2015, p. 122).

### Online behavioral experiment

#### Participants

The original sample consisted of 23 participants, who were recruited from College of Foreign Languages, Henan University. They volunteered to participate in the experiment and were compensated 30 RMB for their participation. All of the participants were native speakers of Chinese. None of them had participated in the norming study. Three of the participants had to be removed from final analyses due to low accuracy rates on literal or anomalous sentences (lower than 70%). The final sample included 20 participants (*M*_age_ = 23.3, *SD*= 1.59; nine males). All the participants had normal or corrected to normal vision. They were all right-handed and none of them have reported any physiological or neurological disorder. Ethical procedures were as in Norming Study 1.

#### Stimuli

Altogether the 120 novel metaphors, 120 novel similes, 120 literal, and 120 anomalous sentences were employed in the experiment. All of the sentences were divided into six blocks, with 20 novel metaphors, 20 novel similes, 20 literal sentences and 20 anomalous sentences in each block. Additionally, 80 filler sentences were added to each block. The filler sentences differed syntactically from the experimental stimuli. To balance out the total number of meaningful and meaningless sentences, in each block, 20 of the filler sentences were meaningful and 60 were meaningless. Each participants completed three blocks. The presentation of the blocks were randomized and participants were not presented with novel metaphors and novel similes that shared the same topic and vehicle.

#### Task

The stimuli were presented centrally in black *Song* font (Size 40) against a gray background using the E-Prime 2.0 software. The sentences were randomly presented word by word. Each trial began with the fixation cross that lasted for 500 ms, followed by a blank screen for 350 ms, after which each word of the sentence was presented for 500 ms. The interval between two consecutive words was 350 ms. The critical word of each sentence ended with a full stop, after which a blank screen appeared (2,000 ms), during which participants could still respond.

Participants were asked to decide whether the presented sentence was meaningful or meaningless by pressing a corresponding key (ENTER vs. CTRL). The response keys were counterbalanced. Before the experiment proper, participants completed a practice session with 20 stimuli not included in the experimental trials.

#### Data analysis

Accuracy rates were calculated as the percentage of correct responses in the semantic decision task. Whether the presented stimulus should be judged as meaningful is based on the results of the norms, namely how meaningful the sentence is on the meaningfulness scale. A repeated measures *ANOVA* with sentence type (novel metaphors vs. novel similes vs. literal sentences vs. anomalous sentences) as within-subject factors showed a main effect of sentence type, *F*_(3,57)_ = 45.39, *p* < 0.001, ηp2 = 0.705. Pairwise comparisons further showed that novel metaphors [*M* = 56.55, *SE* = 3.97, 95% CI (48.24, 64.86)] were judged less accurately than novel similes [*M* = 68.30, *SE* = 3.69, 95% CI (60.58, 76.02)], *p* = 0.003, than literal [*M* = 96.40, *SE* = 0.97, 95% CI (94.37, 98.43)], *p* < 0.001, as well as than anomalous sentences [*M* = 90.00, *SE* = 2.09, 95% CI (85.61, 94.39)], *p* < 0.001. Furthermore, novel similes were judged less accurately than literal, *p* < 0.001, and than anomalous sentences, *p* = 0.002 There was no statistically significant difference between literal and anomalous sentences, *p* = 0.111.

Reaction times (RTs) were measured time-locked to the onset of the final word (critical word) of each utterance type. Only correct responses were used in the RT analysis. A repeated measures *ANOVA* with sentence type (novel metaphors vs. novel similes vs. literal sentences vs. anomalous sentences) as within-subject factors revealed a main effect of sentence type, *F*_(3,57)_ = 47.73*, p*<*0.0*01, $\eta_{\text{p}}^{2}$ = 0.715. Pairwise comparisons showed that novel metaphors [*M* = 1193.21, *SE* = 52.60, 95% CI (1083.12, 1303.31)] evoked longer RTs than literal [*M* = 907.31, *SE* = 39.25, 95% CI (825.15, 989.47)], *p* < 0.001, as well as than anomalous sentences [*M* = 1072.53, *SE* = 49.14, 95% CI (969.67, 1175.39)], *p* = 0.005. Also, novel similes [*M* = 1169.58, *SE* = 52.48, 95% CI (1059.74, 1279.42)] elicited longer RTs than literal, *p* < 0.001, and than anomalous sentences, *p* = *0.0*26. Additionally, anomalous sentences evoked longer RTs relative to literal sentences, *p* < 0.001. Finally, there was no significant difference between novel nominal metaphors and novel similes, *p* = *0.5*72.

## Discussion and conclusions

The present paper aimed to provide norms for novel metaphors, novel similes, literal sentences and anomalous sentences in Chinese, and to show whether comparison mechanisms initiated by a form of a simile might facilitate novel metaphor processing in non-alphabetic languages, such as Chinese.

Results obtained from the norming studies showed that novel metaphors were rated as more meaningful compared to anomalous sentences. Such results indicated that although there is no firm semantic/pragmatic criterion to distinguish novel metaphors from anomalous sentences, anomalous sentences imply greater absurdity (i.e., world knowledge violation) as compared to novel metaphors.

Additionally, novel similes were rated as more meaningful than novel metaphors. Such results were further confirmed by accuracy rates results obtained from the online behavioral experiment. Namely, accuracy rates showed that novel similes were easier to be judged as meaningful compared to novel metaphors. This indicated that novel similes were much easier to comprehend than novel metaphors (Bowdle and Gentner, 2005), as observed in previous studies (Bowdle and Gentner, 2005; Shibata et al., 2012; Lai and Curran, 2013; Jankowiak, 2020; Jankowiak et al., 2021). Nevertheless, reaction time (RT) results revealed that there was no significant differences between novel metaphors and novel similes. Though this result was not in line with the previous studies (Bowdle and Gentner, 2005; Lai and Curran, 2013) showing that novel similes were faster to process than novel metaphors, such a pattern might result from the similarity between novel metaphors and novel similes in Chinese. Namely, in Chinese, both “是” (Eng. *is*) in metaphors and “像” (Eng. *is like*) in similes function as a verb. Thus, novel similes and novel metaphors are very much similar in terms of their morphosyntactic properties. Such similarity between novel metaphors and novel similes might have decreased the differences between these two conditions. Our results might therefore indicate that the facilitating effect of novel similes as postulated by the Career of Metaphor Model (Bowdle and Gentner, 2005) might be sensitive to language-specific features.

The stimuli in the present paper were normed both at the word and sentence level. At the word level, the critical words (sentence-final words) of all the sentence types were matched on their frequency per million, concreteness and number of characters. In addition, at the sentence level, the stimuli were well controlled for in terms of a number of major factors: meaningfulness, familiarity, metaphoricity, and cloze probability. Given that the stimuli have been normed extensively on all the above-mentioned dimensions, they are ideally suited for behavioral and time-related potential (ERP) studies on novel metaphoric and literal language processing, since in these studies, reaction times and/ or ERP amplitudes are time-locked to the onset of the sentence-final word (critical word). In this way, any observed differences elicited by different conditions can only be accounted for by the differences between sentence types, for example, metaphors, literal and anomalous sentences (Jankowiak, 2020).

The aim of the present study was to provide a dataset of novel metaphors, novel similes, literal and anomalous sentences in Chinese. All the sentences were normed for the psycholinguistic variables taken as important in the literature on figurative language processing, namely cloze probability, meaningfulness, metaphoricity and familiarity. An online experiment was conducted in the present study using these stimuli. Results from the experiment lend partial support to the view that comparison mechanisms initiated by similes facilitate novel metaphor comprehension. The final dataset of novel metaphors, novel similes, literal and anomalous sentences are matched for sentence length, cloze probability, meaningfulness, familiarity and metaphoricity. Additionally, the critical words (sentence-final words) are matched for frequency and number of characters. Therefore, the present stimuli can be employed to investigate the online processing of metaphors as well as to test models and theories of metaphor comprehension.

## Data availability statement

The original contributions presented in the study are included in the article/Supplementary material, further inquiries can be directed to the corresponding author.

## Ethics statement

The studies involving human participants were reviewed and approved by Ethics Committee of Psychology and Cognitive Sciences of Henan University. The patients/participants provided their written informed consent to participate in this study.

## Author contributions

XW contributed to conception and design of the study, collection and analysis of the data, and writing and revision of the manuscript.

## Funding

This research was funded by Teaching Reform Research Project in 2020 of Henan University “Exploration of English Writing Teaching Mode for English Majors under the Guidance of the Conceptual Metaphor Theory”.

## Conflict of interest

The author declares that the research was conducted in the absence of any commercial or financial relationships that could be construed as a potential conflict of interest.

## Publisher's note

All claims expressed in this article are solely those of the authors and do not necessarily represent those of their affiliated organizations, or those of the publisher, the editors and the reviewers. Any product that may be evaluated in this article, or claim that may be made by its manufacturer, is not guaranteed or endorsed by the publisher.
