# Supplementary material for: Normed dataset for novel metaphors, novel similes, literal and anomalous sentences in Chinese
Source: Front Psychol. 2022 Sep 1;13:922722. doi: 10.3389/fpsyg.2022.922722 (PMC9477117; doi:10.3389/fpsyg.2022.922722)
Supplement: Supplementary file 2 [file Data_Sheet_2.docx]

**Instructions in Norming Study** 1 and **Norming Study 2**

(Original Chinese, English translations in italics.)

(after Jankowiak, 2019)

**Norming Study 1**

1. 具体度评定

根据1-7级量表，请判断以下名词的具体等级：

1. 极其抽象

2- 非常抽象

3- 相对抽象

4- 既不抽象又不具体

5- 相对具体

6- 非常具体

7- 极其具体

例如,

愤怒 – 1 (非常抽象)

钢笔 – 7 (非常具体)

*Concreteness ratings:*

*On the scale from 1 to 7, please rate how concrete, in your opinion, a particular noun is.*

*1- Definitely abstract*

*2- Mostly abstract*

*3- Rather abstract*

*4- Neither abstract nor concrete*

*5- Rather concrete*

*6- Mostly concrete*

*7- Definitely concrete*

*For instance,*

*anger – 1 (highly abstract)*

*pen – 7 (highly concrete)*

**Norming Study 2**

1. 意义度评定

根据1-7级量表，请判断以下句子的意义度：

1. 完全无意义

2- 无意义

3- 相对无意义

4- 无法判断

5- 相对有意义

6- 有意义

7- 非常有意义

例如，

我的电视机是猪肉 – 1 （完全无意义）

这个女人是教师 – 7（非常有意义）

*Meaningfulness ratings:*

*On the scale from 1-7, please rate how meaningful you think a particular sentence is.*

*1- Totally meaningless*

*2- Meaningless*

*3- Rather meaningless*

*4- Neither meaningless nor meaningful*

*5- Rather meaningful*

*6- Meaningful*

*7- Totally meaningful*

*For instance,*

*My TV is pork – 1 (totally meaningless)*

*This woman is a teacher – 7 (totally meaningful)*

1. 熟悉度评定

根据1-7级量表，请判断你碰到以下句子的经常性：

1. 极少碰到
2. 很少碰到
3. 较少碰到
4. 有时碰到
5. 较多碰到
6. 频繁碰到
7. 极其频繁碰到

例如，

这篇课文是一张桌子 – 1 （极少碰到）

他的父亲是个画家 – 7 （极其频繁碰到）

*Familiarity ratings:*

*On the scale from 1-7, decide how often you encounter a particular sentence.*

*1- Very rarely*

*2- Rarely*

*3- Rather rarely*

*4- Sometimes*

*5- Rather frequently*

*6- Frequently*

*7- Very frequently*

*For instance,*

*This lesson is a desk – 1 (very rarely)*

*His father is a painter – 7 (very frequently)*

1. 隐喻度评定

根据1-7级量表，请判断以下句子的隐喻度：

1. 极其直白

2- 直白

3- 稍微直白

4- 既不直白也不绕弯

5- 稍微绕弯

6- 绕弯

7- 极其绕弯

例如，

他是个士兵 – 1 （极其直白）

生活是旅途– 7 （极其绕弯）

*Metaphoricity ratings:*

*On the scale from 1-7, please rate how metaphorical or literal, according to you, a particular sentence is.*

*1- Very literal*

*2- Literal*

*3- Slightly literal*

*4- Neither literal nor metaphorical*

*5- Slightly metaphorical*

*6- Metaphorical*

*7- Very metaphorical*

*For instance,*

*He is a solider – 1 (very literal)*

*Life is a journey – 7 (very metaphorical)*

1. 填充概率测试

对于下面每一个句子，请用你最先想到的一个名词将之补充完整，并保证句子通顺、有意义。

例如，

这个男孩是 .

这张照片是 .

*Cloze probability tests:*

*Please add a noun that first comes to your mind to the presented beginning of a sentence, so that the sentence is semantically meaningful and grammatically correct.*

*For instance,*

*The boy is a .*

*This picture is a .*
